# The Select Committee on Lunatics

**Published:** 1859-10-01

**Authors:** 


### THE SELECT COMMITTEE ON LUNATICS.

If the Parliamentary inquiry into the present state of the Law of Lunacy and condition of the insane in England prove abortive, it certainly will not arise from insufficiency of time for the digestion of the evidence laid before the Select Committee. Our readers will remember that, by the dissolution of the late Parliament, a Select Committee on Lunatics then sitting was brought to a sudden end, and the Committee simply reported the evidence laid before it, which evidence we noticed at length in our last number. So soon as the present Parliament got into working order, another Committee was appointed, consisting, with one exception, of the same members as the former one. The termination of the last session found, however, the labours of the Committee unfinished; and it has again had to content itself with reporting simply the evidence laid before it, and recommending the re-appointment of the Committee when Parliament again meets. This evidence has appeared too recently, and is too voluminous to be dealt with in the present number of our Journal, consequently we are obliged to postpone the consideration of it until a subsequent period.

We would, however, note one point in reference to pauper lunatics, which has an important bearing upon several of the remarks which we made in our article on "Pauper Lunacy," in the last number of the Journal. Mr. A. Doyle, one of the Inspectors under the Poor-Law Board, who gave evidence before the Select Committee, questions generally the truthfulness of the facts and conclusions respecting workhouses and the insane contained in them, recorded in the Supplement to the Twelfth Report of the Commissioners in Lunacy. It will be requisite, in examining the evidence reported by the Select Committee to enter pretty fully into Mr. Doyle's objections, but, in the meantime, we record his dissent, reserving, for the present, any expression of opinion upon it.

